# Off-Centered Pb Interstitials in PbTe

**DOI:** 10.3390/ma15041272

**Published:** 2022-02-09

**Authors:** Sungjin Park, Byungki Ryu, SuDong Park

**Affiliations:** Energy Conversion Research Center, Korea Electrotechnology Research Institute (KERI), Changwon 51543, Korea; sjinpark@keri.re.kr (S.P.); john@keri.re.kr (S.P.)

**Keywords:** PbTe, Pb interstitial, off-centered, symmetry breaking, defect–lattice interaction

## Abstract

Previous calculations have demonstrated that Te vacancies are energetically the major defects in PbTe. However, the Pb interstitials are also important because experiments have shown that the volume of Pb-rich PbTe increases at a higher Pb content. In this study, density functional theory calculations were used to investigate the defect properties of low-symmetry Pb interstitials in PbTe. By breaking the higher symmetry imposed on the on-centered interstitial defects, the lowest ground state of Pb interstitial defects is off-centered along the $\left[\bar{1}\bar{1}\bar{1}\right]$ direction. Because of the four multi-stable structures with low defect-formation energies, the defect density of Pb interstitials is expected to be approximately six times higher than previous predictions for PbTe synthesized at 900 K. In contrast to the on-centered Pb interstitials, the off-centered Pb interstitials in PbTe can exhibit long-range lattice relaxation in the [111] direction beyond a distance of 1 nm, indicating the potential formation of weak local dipoles. This result provides an alternative explanation for the emphanitic anharmonicity of PbTe in the high-temperature regime.

## 1. Introduction

Thermoelectric effects enable the direct conversion between thermal and electrical energies [1]. The thermoelectric conversion efficiency can be estimated using the dimensionless figure of merit ZT = α^2^σT/(κ_elec_ + κ_latt_), where α is the Seebeck coefficient, σ is the electrical conductivity, κ_elec_ and κ_latt_ are the electronic and lattice thermal conductivities, respectively, and T is the absolute temperature [1,2]. Because a large ZT value can lead to higher thermoelectric efficiency, reducing the lattice thermal conductivity and optimizing the thermoelectric power factor (α^2^σ) have been key strategies for developing materials with high thermoelectric performance [1,3,4]. For example, thermoelectric performance can be improved by introducing imperfections into well-ordered lattices, introducing a second phase by extrinsic defects in disordered lattices, and nanoengineering [5,6]. This is because these strategies increase the electrical conductivity without changing the carrier mobility, reduce the thermal conductivity by phonon scattering, and enhance the Seebeck coefficient by controlling the density of states and charge carrier scattering.

PbTe-based materials have high thermoelectric performance for middle-temperature applications up to a hot-side temperature of 800 K [7,8,9,10,11,12,13]. Doping with Na, Bi, Cr, or I, introduces suitable charge carriers and moves the Fermi level near the optimal band edge positions, thus optimizing the power factor [14,15,16,17,18]. Alloying or doping with extrinsic Ag, Sb, CdTe, MgTe, MnTe, SrTe, EuTe, or Ag_2_Te phases leads to low lattice thermal conductivities near or below 1 W/m/K [7,8,10,11,19,20,21,22]. As a result, PbTe-based thermoelectric materials with ZT greater than 2.5 have been reported [21,23,24,25]. For example, Na_0.02_Pb_0.98_Te-8% SrTe showed a ZT of 2.5 at 923 K, due to an increased power factor and a decreased lattice thermal conductivity from converging valence bands and widening the band gap through alloying with SrTe [21]. In addition, the ZT of Na_0.03_Eu_0.03_Sn_0.02_Pb_0.92_Te reached 2.51 at 823 K through lattice strain control due to Pb substitutional defects (Na_Pb_, Eu_Pb_, and Eu_Sn_) [23].

In addition to further enhancing the ZT value of PbTe by alloying or doping studies, researchers have also proposed several intrinsic mechanisms for this material’s high thermoelectric performance. The complex low-energy band structure of PbTe with non-parabolicity and high valley degeneracy is responsible for the band convergence and high thermoelectric power factor α^2^σ [9]. Meanwhile, the anharmonic phonon structure has been considered the origin of its low thermal conductivity. While there are no off-centered Pb atoms in the PbTe sublattices [26,27,28], Pb atoms can show large thermal displacements at high temperatures, indicating strong phonon anharmonicity [27,29,30,31]. More recently, PbTe was reported to exhibit local symmetry breaking upon heating, which is referred to as *emphanisis* [32]. Although the space- and time-averaged structures of PbTe show a high-symmetry on-centered lattice [32], single-crystal X-ray diffuse scattering analysis accompanied with ab initio molecular dynamics revealed the formation of local dipoles extending over a few unit cells, and the time evolution of these local dipoles was estimated to be approximately 10 ps. This *emphanitic behavior* of local dipole fluctuations at high temperatures is different from the normal phase transition observed in GeTe, Cu_2_Te, and Ag_2_Te, where low-symmetry globally polarized phases are stabilized below the phase transformation temperature [33,34,35,36,37].

Meanwhile, the nature of intrinsic defects in PbTe has been investigated. A first-principles study of PbTe predicted that a Pb vacancy (V_Pb_) is a shallow acceptor state while a Te vacancy (V_Te_) forms a deep donor state, and Schottky defects generate donor–acceptor pairs inside the band gap [38]. In addition, molecular dynamics simulations have shown that the lattice thermal conductivity of PbTe can be reduced by intrinsic point defects [39]. More recent first-principles calculations revealed that PbTe has several intrinsic defects because the charged defect-formation energies of intrinsic defects are small (<1 eV) [40,41,42,43]. The point intrinsic defects pictures accurately describe the electrical properties of binary phases. Under the Pb-rich condition, PbTe is known to exhibit *n*-type conduction behavior due to the shallow $V_{\mathrm{Te}}$ donor, while under the Te-rich condition, PbTe shows *p*-type conduction due to the shallow $V_{\mathrm{Pb}}$ acceptor.

Interestingly, previously predicted defect formation energies under Pb-rich conditions [41,42] are inconsistent with the observed lattice expansion in PbTe [43,44]. Experimental analysis showed that the lattice parameter of binary PbTe changes depending on the ratio of Pb and Te in PbTe [43,44]. Under Pb-poor conditions, the lattice parameter decreased as the ratio of Pb deficiency (compared to that of PbTe) increased [44]. Under Pb-rich conditions, the lattice volume increased as the ratio of excess Pb (also compared to that of PbTe) increased [44]. In addition, the Pb-rich condition enlarged the lattice parameter of binary PbTe by up to approximately 0.3% when the ratio of excess Pb reached approximately 8% [43]. This can be attributed to intrinsic defects because PbTe is binary. Te vacancy as a major defect in Pb-rich PbTe can hardly explain these lattice parameter changes because vacancy defects generally reduce the lattice volume instead. Thus, previously reported defect formations under Pb-rich conditions [41,42] simply cannot explain the experimentally observed lattice expansion of PbTe [43,44].

Overall, there is a lack of information on the relationship between the existence of intrinsic defects and the PbTe lattice. Although interstitial defects can increase the lattice volume of PbTe, the most stable defect is not the Pb interstitial ($I_{\mathrm{Pb}}$) but $V_{\mathrm{Te}}$ according to density functional calculations. To address the discrepancy between the theoretically predicted defect formation energy and the experimentally observed lattice parameter increase, a defect cluster model was used to explain lattice expansion [43]. Although the difference in defect formation energy between $I_{\mathrm{Pb}}$ and $V_{\mathrm{Te}}$ is high and cannot be neglected, non-equilibrium synthesis of the material can create high-energy defects. Moreover, the previous investigations were based on a well-defined high-symmetry configuration [41,42], which neglects possible interactions between intrinsic defects and the host PbTe lattice.

In this study, first-principles calculations were performed to further elucidate the interactions between intrinsic defects and the Pb-rich PbTe lattice. First, the charged defect formation energies of intrinsic defects in binary PbTe are revisited, particularly for $I_{\mathrm{Pb}}$. Because the PbTe lattice is reported to be soft [27], possible low-symmetry configurations for single atomic intrinsic defects (vacancy, interstitial, antisite) are carefully investigated by breaking the symmetry of the high-symmetry configurations. Moreover, taking the experimental results [43,44] to be accurate, it was assumed that the $I_{\mathrm{Pb}}$ defects may be responsible for the increased lattice parameter and those were considered as the dominant intrinsic defects inside Pb-rich PbTe. The $I_{\mathrm{Pb}}$ defects have been intensively investigated because they can enlarge the lattice volume, unlike other types of defects such as $V_{\mathrm{Te}}$. In addition, low-symmetry $I_{\mathrm{Pb}}$ significantly affects the atomic positions of the host lattice. Thus, focusing on high-symmetry and low-symmetry $I_{\mathrm{Pb}}$, this study examines the energetics of intrinsic defects, the structural difference between various defective lattices, and the interactions between defects and the host lattice.

## 2. Computational Methods

First-principles density functional theory (DFT) calculations [45,46] were performed using the Vienna Ab initio simulation package (VASP.5.4.4) [47,48]. We used a plane-wave basis set with an energy cutoff of 320 eV, the Perdew–Burke–Ernzerhof (PBE) exchange-correlational functional [49], and projector augmented wave (PAW) pseudopotentials [50,51] (PAW_PBE Pb_d_GW 14Apr2014, PAW_PBE Te_GW 22Mar2012). A 3×3×3 Γ-centered k-point mesh grid was used for k-space integration in the Brillouin zone. To consider the relativistic effect of heavy elements, spin–orbit interaction (SOI) calculations [51] were used while the defective structures of PbTe were relaxed.

The lattice parameter of PbTe (6.5758 Å) was obtained by Murnaghan fitting [52,53]. Note that this calculated value is slightly larger (by 1.7%) than the experimentally measured value of 6.462 Å at room temperature [27]. Such a small overestimation of lattice parameter is a well-known error in generalized gradient approximation (GGA) calculations. Because PbTe has one of the largest lattice parameters among the materials, lattice overestimation can affect the structural relaxation of defects. A previous experimental study found that the lattice parameter can change by up to ~2% when the temperature is changed from 0 to 900 K [27]. Thus, our optimized lattice parameter is applicable to the high-temperature behavior of PbTe, although it is larger than the value at 0 K for PbTe.

A 128-atom fcc supercell was used to investigate the defect structure of PbTe. All possible intrinsic point defects, namely the interstitials, vacancies, and antisites, were considered. To create a defective supercell, one extra atom was added to the supercell for interstitials, one original atom inside the supercell was removed for vacancies, and one original cation (or anion) inside the supercell was replaced with a counterpart anion (or cation) for antisites. Then, the defect structures were relaxed until the remaining forces were smaller than 10^−4^ eV/Å. Note that a low atomic force criterion is critical for determining the atomic structures of defective PbTe, owing to the long-range interactions between the atoms in PbTe [54]. In particular, various low-symmetry configurations of intrinsic defects in the supercell were explored by checking the configuration stability between perturbed structures. Finally, the interstitial defect configurations were categorized into stable, unstable, and saddle configurations.

Regarding the size and the shape of the supercell used in our calculations, it is important to consider a computational supercell that is large enough and has an appropriate shape. Owing to the computational costs, our calculations were performed in a 4 × 4 × 4 128-atom fcc supercell. Our supercells might show the size effect because of their limited volumes. However, it is important to consider the various symmetry-breaking positions of I_Pb_ in a slightly smaller supercell, rather than using a sufficiently large supercell to completely exclude the size effect.

The properties of point intrinsic defects were investigated at various charge states: 2+, 0 (neutral), and 2−. The formation energy ($E_{\mathrm{Form}}\left[D^{q}\right]$) was calculated as follows:

(1) $$E_{\mathrm{Form}}\left[D^{q}\right]={\;E}_{\mathrm{Tot}}\left[D^{q}\right]-E_{\mathrm{Tot}}\left[\mathrm{PbTe}\right]+\sum_{i}{\Delta n}_{i}\mu_{i}+qE_{\mathrm{Fermi}}\;,$$

where $E_{\mathrm{Tot}}\left[D^{q}\right]$ is the total energy of the defective PbTe supercell in the charge state of *q*, and $E_{\mathrm{Tot}}\left[\mathrm{PbTe}\right]$ is the total energy of pristine PbTe [40,55]. In Equation (1), for a specific atom *i* (Pb or Te) inside the supercell, ${\Delta n}_{i}$ and $\mu_{i}$ represent the change in the number of this type of atom in the supercell and its atomic chemical potential, respectively. $E_{\mathrm{Fermi}}$ is the electron Fermi level of the host PbTe [40].

In addition, the equilibrium defect density ($n_{D}$) is defined as:

(2) $$n_{D}=N_{\mathrm{site}}\;\exp\left(-E_{\mathrm{Form}}\left[D^{q}\right]\;/\;k_{B}T\;\right),$$

where $N_{\mathrm{site}}$ is the number of available sites for defects, and $k_{B}$ is the Boltzmann constant. From this equation, the ratio of two defect densities (nD1nD2) can be defined as:

(3) nD1nD2=Nsite,D1Nsite,D2 exp (−ΔEForm / kBT ), 

where $\Delta E_{\mathrm{Form}}=E_{\mathrm{Form}}\left[D_{1}^{q}\right]-E_{\mathrm{Form}}\left[D_{2}^{q}\right]$ is the difference in the formation energies between defects $D_{1}^{q}$ and $D_{2}^{q}$.

## 3. Results and Discussions

**Figure 1:**
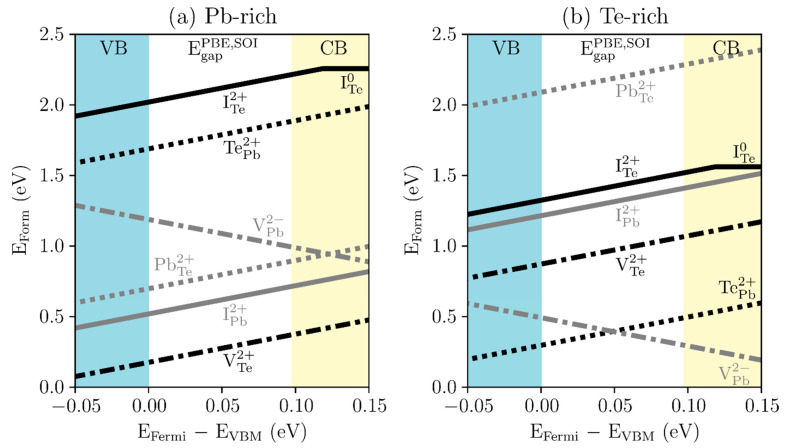
Charged defect-formation energies (E_Form_) of high-symmetry defect configurations in PbTe under the (**a**) Pb-rich and (**b**) Te-rich conditions: Te vacancy (V_Te_: black dot-dashed line), Pb antisite by Te (Te_Pb_: black dotted line), Te interstitial (I_Te_: black solid line), Pb vacancy (V_Pb_: gray dot-dashed line), Te antisite by Pb (Pb_Te_: gray dotted line), and Pb interstitial (I_Pb_: gray solid line). The x-axis represents the difference between energies of the Fermi level (E_Fermi_) and the valence band maximum (E_VBM_). The yellow region indicates the conduction band (CB), the blue region the valence band (VB), and the white region the PBE energy gap with SOI ($E_{\mathrm{gap}}^{\mathrm{PBE},\mathrm{SOI}}$).

First, the formation energies of charged defects for the high-symmetry defect configurations are revisited in PbTe. Figure 1a,b show the charged defect-formation energies in PbTe under Pb- and Te-rich conditions, respectively. $V_{\mathrm{Te}}$, $I_{\mathrm{Pb}}$, and Pb antiste at the Te site (${\mathrm{Pb}}_{\mathrm{Te}}$) are the major defects in Pb-rich PbTe. Under Pb-rich conditions, the formation energy is the lowest for $V_{\mathrm{Te}}$, followed by $I_{\mathrm{Pb}}$ and ${\mathrm{Pb}}_{\mathrm{Te}}$. The Te antisite at the Pb site (${\mathrm{Te}}_{\mathrm{Pb}}$) and Te interstitial ($I_{\mathrm{Te}}$) defects have high formation energies, above 1.688 eV. 

On the other hand, under Te-rich conditions, ${\mathrm{Te}}_{\mathrm{Pb}}$ and $V_{\mathrm{Pb}}$ have the lowest defect-formation energies. These results are generally consistent with previous calculations [41,42,43]. Note that the PBE band gap obtained from SOI calculation is 0.098 eV, which is smaller than the experimentally observed band gap (0.3–0.4 eV) due to the problem of band gap underestimation in DFT calculations. Thus, band gap correction can change the stability order between defects, especially for the Te-rich cases. However, for the Pb-rich case, the relative defect stability does not change with the Fermi level position because the defects with lower formation energies are all 2+ charge states. Thus, for Pb-rich off-stoichiometric PbTe, it is speculated that the defect physics will be less sensitive to the calculation setting or the choice of exchange-correlation functionals that is important for defect charge states.

**Figure 2:**
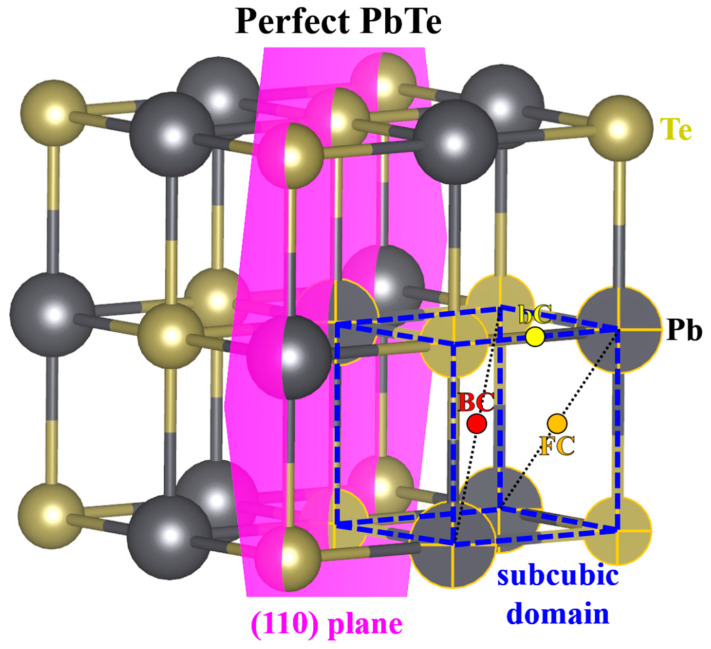
Rock-salt atomic structure of perfect PbTe. The black and gold balls indicate Pb and Te atoms, respectively. The blue dashed lines represent the defined subcubic domain. Its high-symmetry positions consist of the bond center (bC, yellow dot) of Pb−Te, the face center (FC, orange dot), and the body center (BC, red dot). The thin black dotted lines inside the subcubic domain are a guide to the eye for the high-symmetry locations. The violet plane is a (110) plane.

Figure 2 shows the rock-salt structure of pristine PbTe with a lattice parameter of 6.5758 Å. There are two basis positions in the primitive PbTe unit cell: Pb at (0 0 0) and Te at (1/2 1/2 1/2). Pb is surrounded by six first nearest neighbor (FNN) Te atoms, and Te has six FNN Pb atoms. The Pb–Te bond length was calculated to be 3.2879 Å. Here, a small Pb_4_Te_4_ cubic region in PbTe is called a *subcubic domain*, denoted by the blue dashed lines in Figure 2. The conventional cubic unit cell for PbTe contains eight units of the subcubic domain. Although the subcubic domain is not a unit cell, it is geometrically equivalent owing to the point symmetry of the PbTe lattice. Therefore, when searching for single-defect configurations of low-symmetry $I_{\mathrm{Pb}}$ defects, defects in a single subcubic domain were only considered.

To find the local potential minima and metastable states, all possible irreducible positions of $I_{\mathrm{Pb}}$ were looked for. Note that vacancies and antisite defects can also be off-centered. However, $I_{\mathrm{Pb}}$ defects are intensively investigated because they could enlarge the lattice volume, unlike other types of defects such as $V_{\mathrm{Te}}$. There are three distinct high-symmetry positions for interstitials in the supercell: the center of the Pb–Te bond, the plane center of the subcubic domain, and the body center of the subcubic domain (BC). To break the symmetry of the defective subcubic domain, the following configurations perturbed from high-symmetry positions were considered. (1) $I_{\mathrm{Pb}}$ at the bond center can be perturbed in the FNN Pb direction, FNN Te direction, and two normal directions of the bond (toward BC and the adjacent plane center). Thus, $I_{\mathrm{Pb}}$ at the bond center has only four perturbed configurations. (2) $I_{\mathrm{Pb}}$ at the plane center can be displaced in the direction of two types of FNN atoms (Pb and Te). It can also be disturbed in the normal direction of the plane (equivalent to the direction of BC from the plane center) and in the direction perpendicular to the BC direction (equivalent to the direction of the adjacent bond center from the plane center). Therefore, $I_{\mathrm{Pb}}$ at the plane center also has only four perturbed configurations. (3) $I_{\mathrm{Pb}}$ at BC can be dislocated in four directions: the directions to two distinguishable FNN atoms (Pb and Te), the direction to the FNN plane center of the subcubic domain, and the direction to the FNN bond center of the subcubic domain. Note that because of the geometry of the subcubic domain, the four FNN Pb (or Te) directions from the BC are equivalent, the six FNN plane-center directions from the BC have the same symmetry, and the twelve FNN bond-center directions from the BC are equivalent geometrical directions. Consequently, even $I_{\mathrm{Pb}}$ at the BC has only four perturbed configurations. Considering the above $I_{\mathrm{Pb}}$ positions that include both the high-symmetry ones and symmetry-broken low-symmetry ones, structural relaxation for 15 irreducible $I_{\mathrm{Pb}}$ positions were performed.

Three critical configurations for the five BC-related $I_{\mathrm{Pb}}$ configurations were identified. For $I_{\mathrm{Pb}}$ positions related to the bond center and the plane center of the subcubic domain, defect formation energies of the 2+ charge state are higher (by 1.6 and 0.7 eV, respectively) than that of $I_{\mathrm{Pb}}$ located at the BC-related high-symmetry position ($I_{\mathrm{Pb}}^{\mathrm{on}}$). Based on their stability and structural features, three local extrema, namely $I_{\mathrm{Pb}}^{\mathrm{on}}$, BC-related $I_{\mathrm{Pb}}$ with a slight displacement in the direction of one FNN Pb atom ($I_{\mathrm{Pb}}^{\mathrm{off}}$), and BC-related $I_{\mathrm{Pb}}$ with a large movement toward one FNN Pb to form a Pb–Pb dimer ($I_{\mathrm{Pb}}^{\dim}$), were selected. Note that $I_{\mathrm{Pb}}^{\mathrm{on}}$ is located at the center of the subcubic domain, and $I_{\mathrm{Pb}}^{\mathrm{off}}$ is slightly displaced from the center to one of the four nearest host Pb atoms. In addition, $I_{\mathrm{Pb}}^{\dim}$ indicates the formation of a Pb dimer by farther moving I_Pb_ toward one of the four nearest Pb atoms. Note that $I_{\mathrm{Pb}}^{\mathrm{on}}$ is not a stable defect, but $I_{\mathrm{Pb}}^{\mathrm{off}}$ is the ground state configuration for $I_{\mathrm{Pb}}$. Furthermore, $I_{\mathrm{Pb}}^{\dim}$ is the saddle configuration and has a larger formation energy than $I_{\mathrm{Pb}}^{\mathrm{on}}$.

**Figure 3:**
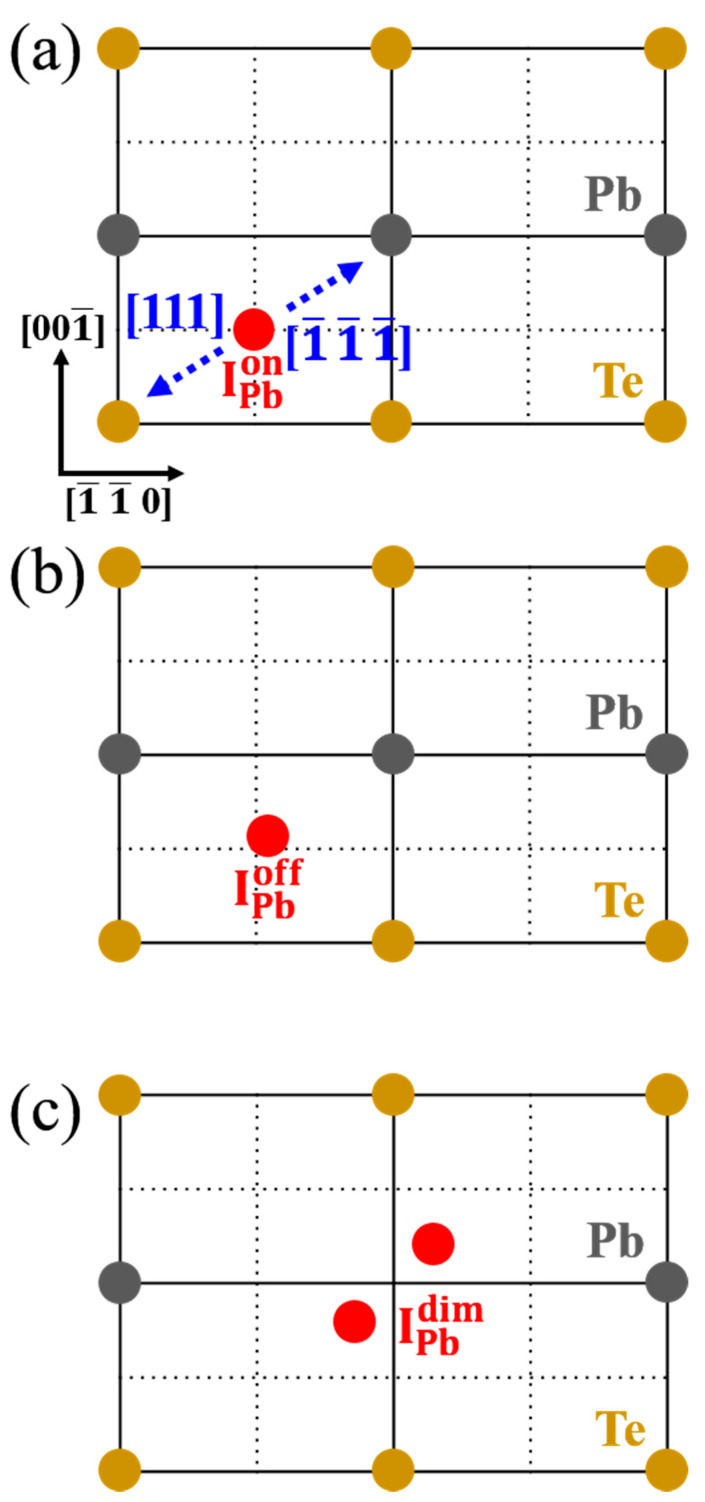
Schematic atomic structures projected to the (110) plane of Pb interstitial at (**a**) the on-centered position ($I_{\mathrm{Pb}}^{\mathrm{on}}$), (**b**) the off-centered position ($I_{\mathrm{Pb}}^{\mathrm{off}}$), and (**c**) the Pb-dimer ($I_{\mathrm{Pb}}^{\dim}$) position. $I_{\mathrm{Pb}}^{\mathrm{on}}$ is located at the center of the subcubic domain, and $I_{\mathrm{Pb}}^{\mathrm{off}}$ is slightly displaced from the center of the subcubic domain to one of the four nearest host Pb atoms. In addition, $I_{\mathrm{Pb}}^{\dim}$ involves the formation of a Pb dimer by farther moving of a Pb interstitial toward one of the four nearest Pb atoms. Each red dot indicates a Pb interstitial defect. The black and gold dots represent Pb and Te atoms, respectively. The black solid lines represent the (110) planes of the PbTe lattice, and the black dotted lines are a guide to the eye for the center of the subcubic domain. The blue arrows indicate the directions toward the nearest neighbor Pb ([$\bar{1}\bar{1}\bar{1}$]) and the nearest neighbor Te ([111]) of Pb interstitial defect.

Figure 3a–c shows schematic atomic structures of the three important interstitial defects found here, namely $I_{\mathrm{Pb}}^{\mathrm{on}}$, $I_{\mathrm{Pb}}^{\mathrm{off}}$, and $I_{\mathrm{Pb}}^{\dim}$, respectively. In Figure 3a, $I_{\mathrm{Pb}}^{\mathrm{on}}$ is located at the center of the subcubic domain, which is one of the high-symmetry positions. This position has four FNN Pb atoms and four FNN Te atoms. Figure 3b,c display the structures induced by symmetry breaking: one is slightly displaced in the direction of an FNN Pb along $\left[\bar{1}\bar{1}\bar{1}\right]$, while the other is farther displaced toward a Pb along $\left[\bar{1}\bar{1}\bar{1}\right]$. The latter configuration can be formed only when $I_{\mathrm{Pb}}$ forms a symmetric Pb–Pb dimer structure, which is a saddle point. Otherwise, the $I_{\mathrm{Pb}}$ atom relaxes back toward the $I_{\mathrm{Pb}}^{\mathrm{off}}$ position.

**Figure 4:**
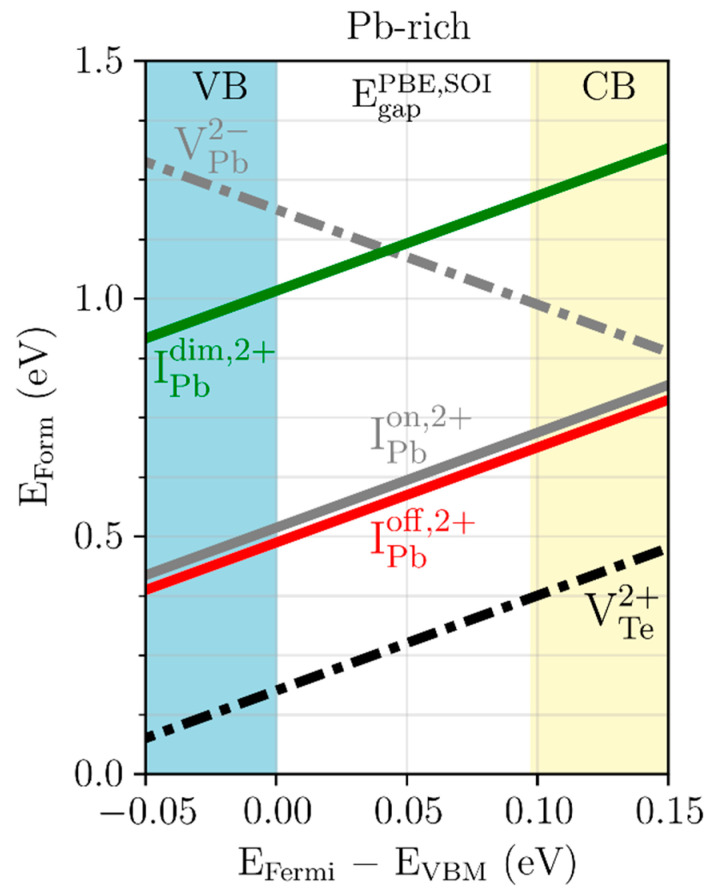
Defect formation energy (E_Form_) under the Pb-rich condition of the Pb vacancy ($V_{\mathrm{Pb}}^{2-}$: gray dot-dashed line), Te vacancy ($V_{\mathrm{Te}}^{2+}$: black dot-dashed line), as well as the on-centered ($I_{\mathrm{Pb}}^{\mathrm{on},2+}$: gray solid line), off-centered ($I_{\mathrm{Pb}}^{\mathrm{off},2+}$: red solid line), and Pb-dimer ($I_{\mathrm{Pb}}^{\dim,2+}$: green solid line) Pb interstitial defects. The x-axis represents the difference between the Fermi level (E_Fermi_) and the valence band maximum energy ($E_{\mathrm{VBM}}$). The yellow region indicates the conduction band (CB), the blue region the valence band (VB), and the white region the PBE energy gap calculated with SOI ($E_{\mathrm{gap}}^{\mathrm{PBE},\mathrm{SOI}}$).

In Figure 4, effects of symmetry breaking on the defect stability of $I_{\mathrm{Pb}}^{\mathrm{on}}$, $I_{\mathrm{Pb}}^{\mathrm{off}}$, and $I_{\mathrm{Pb}}^{\dim}$ were first investigated. Note that Figure 1a includes only the formation energy of $I_{\mathrm{Pb}}^{\mathrm{on}}$ for Pb interstitials, whereas Figure 4 shows those of all three types of Pb interstitials depending on their positions. All three defects are shallow donors as they are stable in the 2+ charge state in the entire range of the energy gap. $I_{\mathrm{Pb}}^{\mathrm{off}}$ is the most stable defect among Pb interstitials because it has the lowest formation energy except for V_Te_. Although the difference in formation energy is small, their structural difference is significant, as will be discussed below. The difference in formation energy between $I_{\mathrm{Pb}}^{\mathrm{off}}$ and $I_{\mathrm{Pb}}^{\mathrm{on}}$ is 0.032 eV when the defect charge state is 2+. For the neutral charge state, this difference is reduced to 0.004 eV. When they are negatively charged, a negligible energy difference (~0.0003 eV) is found between the off-centered and on-centered ones. These charge state-dependent energetics imply that $I_{\mathrm{Pb}}^{\mathrm{off}}$ only appears when the defects are positively charged. On the other hand, $I_{\mathrm{Pb}}^{\dim}$ has a much larger formation energy than the on-centered $I_{\mathrm{Pb}}^{\mathrm{on}}$. In the 2+ charge state the energy difference is 0.498 eV, which is slightly reduced to 0.382 and 0.241 eV in the neutral and 2− charge states, respectively. It is emphasized that this is the first report of off-centered interstitial defects in PbTe, while only high-symmetry configurations have been reported before [41,42,43].

To further understand the results of Figure 4, the formation energies were analyzed for the charged defects $V_{\mathrm{Te}}^{2+}$, $I_{\mathrm{Pb}}^{\mathrm{off},2+}$, $I_{\mathrm{Pb}}^{\mathrm{on},2+}$, and $I_{\mathrm{Pb}}^{\dim,2+}$. The positively charged $V_{\mathrm{Te}}^{2+}$ defect state is the most stable intrinsic defect in Pb-rich PbTe. Meanwhile, the formation energy of high-symmetry $I_{\mathrm{Pb}}^{\mathrm{on},2+}$ defects only differs by approximately 0.441 eV from that of the high-symmetry $V_{\mathrm{Te}}^{2+}$. By breaking the high symmetry, the interstitial is slightly displaced in the direction of one of the four FNN Pb atoms or in other equivalent directions, leading to a reduction in the formation energy difference by 0.032 eV. Moreover, the on-centered $I_{\mathrm{Pb}}^{\mathrm{on},2+}$ site is unstable. Beyond the off-centered interstitial configuration, it was found that the Pb–Pb dimer interstitial configuration has a higher defect formation energy than the on-centered one. On the other hand, in contrast to the off-centered structure, the on-centered interstitial and Pb–Pb dimer type defects are unstable or have saddle point configurations. Despite their unstable nature, these can serve as *intermediate* states between interstitial configurations while the Pb atoms diffuse.

Note that $I_{\mathrm{Pb}}^{\mathrm{on}}$ has only one available site. However, because there are four possible defect sites with lower defect formation energies for $I_{\mathrm{Pb}}^{\mathrm{off}}$, there can be more interstitial defects compared to the previous prediction. If it is assumed that the defects are generated at a temperature of 900 K and the formation energy difference is 0.032 eV, then the density ratio between the off-centered interstitials and the on-centered ones is 6.04 according to Equation (3). Thus, symmetry breaking can enhance the defect density by 504%.

Next, the effects of $I_{\mathrm{Pb}}$ on the atomic structure of PbTe were investigated. To measure the structural change of each atom in the supercell, the structural relaxation parameter of atom *i* by the defect D was defined as:

(4) $$R_{i}\left(D\right)\equiv \;\left|{\vec{r}}_{i}-{\vec{r_{i}}}^{\left(0\right)}\right|$$

where ${\vec{r}}_{i}$ is the position vector of atom *i* in the supercell after structural relaxation by the $I_{\mathrm{Pb}}$ defect. ${\vec{r_{i}}}^{\left(0\right)}$ is the position vector of atom *i* before structural relaxation, that is, the original atomic position in pristine PbTe. The distance parameter d of atom *i* from the defect D was also defined as:

(5) $$d_{i-D}\equiv \;\left|{\vec{r}}_{i}-{\vec{r}}_{D}\right|$$

For the change in distance, the reference distance $d_{i-D}^{\left(0\right)}$ from the ideal defect position to the ideal atomic position before relaxation was also computed.

Table 1 shows short-range structural relaxation of neighboring host atoms near the $I_{\mathrm{Pb}}$ defects, where the distance $d_{i-D}^{\left(0\right)}$ is smaller than or equal to 7.166 Å. Before structural relaxation, there are 4 Pb FNNs and 4 Te FNNs, 12 Pb second nearest neighbors (SNNs), 12 Te SNNs, 12 Pb third nearest neighbors (TNNs), and 12 Te TNNs at distances of 2.847, 5.452, and 7.166 Å for $I_{\mathrm{Pb}}^{\mathrm{on},2+}$, respectively. After structural relaxation, the distances from the defect to its neighbors change. Note that the distances to the Pb FNNs and Te FNNs increase regardless of the type of $I_{\mathrm{Pb}}$. In the case of $I_{\mathrm{Pb}}^{\mathrm{on},2+}$, structural relaxation slightly increased the distance to the Pb FNNs compared to that to the Te FNNs (3.349 and 3.108 Å, respectively). This difference in structural change may be explained by electrostatic interactions between the charges of the defects and the local environment. The structural relaxation behavior of $I_{\mathrm{Pb}}^{\mathrm{off},2+}$ is distinguished from that of the on-centered defect because the distances from $I_{\mathrm{Pb}}^{\mathrm{off},2+}$ to the FNNs fall into two groups. After breaking the symmetry, $I_{\mathrm{Pb}}$ becomes off-centered, meaning that it moves closer to one of the four FNN Pb atoms and away from the other three atoms. Thus, there exist one shorter bond and three longer bonds. However, the distances from the defect to the four FNN Te atoms are almost the same. Although the structural relaxation parameters $R_{i}\left(D\right)$ of the TNNs are smaller than those of the FNN atoms, the structural change does not vanish even for host atoms beyond the FNN shells. For example, the distances from the defect to the 12 TNN atoms of Pb and Te are not equal in the supercell containing the off-centered defect. The *symmetry-breaking* phenomenon was also clearly observed in the TNN atomic shells of both Pb and Te atoms. In addition, the values of $d_{i-D}$ for TNNs are significantly larger than those for FNNs. Thus, although structural relaxation is the largest for atoms in the FNN shells, it still persists even for atoms far from the off-centered defect.

**Figure 5:**
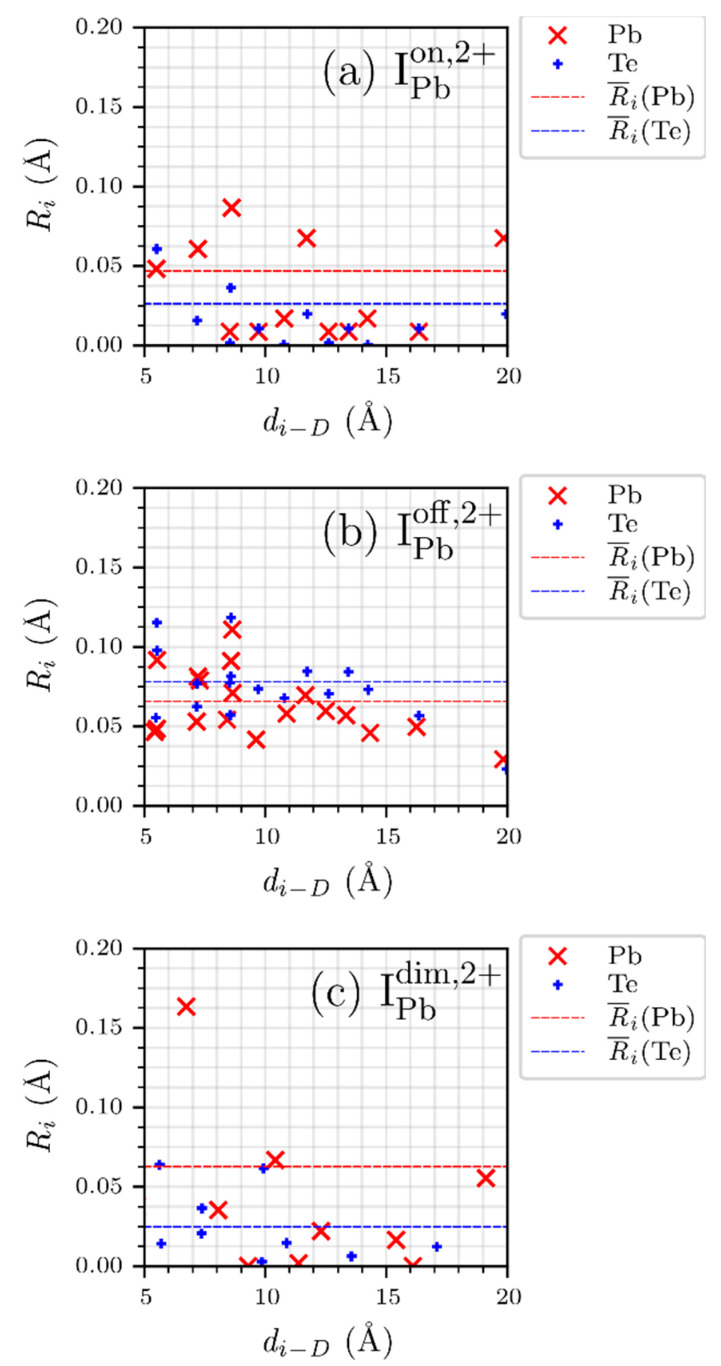
Structural relaxation parameters ($R_{i}$) of host atoms excluding the nearest neighbors of the Pb interstitial defect at the (**a**) on-centered ($I_{\mathrm{Pb}}^{\mathrm{on},2+}$), (**b**) off-centered ($I_{\mathrm{Pb}}^{\mathrm{off},2+}$), and (**c**) dimer ($I_{\mathrm{Pb}}^{\dim,2+}$) locations. Red “×” and blue “+” represent the data for Pb and Te atoms, and the red and blue dashed lines represent the average of $R_{i}$ for Pb and Te, respectively. The x-axis represents the defect distance parameter $d_{i-D}$.

Next, the long-range effects of $I_{\mathrm{Pb}}$ on PbTe were investigated. Figure 5 shows the relationship between the structural relaxation parameter and the defect distance. It is found that the long-range interaction between $I_{\mathrm{Pb}}$ and PbTe is stronger when the defect symmetry is lowered, and the interaction range of $I_{\mathrm{Pb}}^{\mathrm{off},2+}$ exceeds 1 nm. $R_{i}\left(D\right)$ is regarded as non-negligible if it is larger than 0.05 Å. For $I_{\mathrm{Pb}}^{\mathrm{on},2+}$, $R_{i}\left(D\right)$ is not negligible at $d_{i-D}=$ 11.714 Å, whereas it begins to vanish at $d_{i-D}=$ 12.623 Å. In addition, there is no split of $R_{i}\left(D\right)$ over the whole range of $d_{i-D}$. We think that $R_{i}\left(D\right)$ at $d_{i-D}=$ 19.865 Å may be due to the supercell size effect. However, for $I_{\mathrm{Pb}}^{\mathrm{off},2+}$, a significant long-range interaction was observed in the defective supercell. $R_{i}\left(D\right)$ is still 0.057 Å for $d_{i-D}=$ 13.346 Å. Furthermore, there remain clear splits of $R_{i}\left(D\right)$ until $d_{i-D}=$ 8.658 Å, as can be seen in Figure 5b. From Table 1 and Figure 5, it is concluded that the off-centered defect structure is different from the on-centered one. The supercell containing $I_{\mathrm{Pb}}^{\dim,2+}$ also shows clear differences in structural relaxation from the other configurations, as seen in Figure 3 and Figure 5. Owing to $I_{\mathrm{Pb}}^{\dim,2+}$, structural relaxation of Pb atoms in the supercell is significantly larger for the shorter distances than the others. However, the relaxation rapidly decreases compared to that in the off-centered configuration. In addition, the average $R_{i}\left(D\right)$ of the supercell involving $I_{\mathrm{Pb}}^{\dim,2+}$ with respect to Pb and Te is smaller than that of $I_{\mathrm{Pb}}^{\mathrm{off},2+}$ but slightly larger than that of $I_{\mathrm{Pb}}^{\mathrm{on},2+}$. Note that although the atomic structure involving $I_{\mathrm{Pb}}^{\dim,2+}$ is also of low symmetry, it has higher symmetry than the off-centered one.

The effect of the charge state on the structural change between the supercells of $I_{\mathrm{Pb}}^{\mathrm{off}}$ and $I_{\mathrm{Pb}}^{\mathrm{on}}$ was analyzed by computing $\Delta d_{i-D}^{\mathrm{off},\mathrm{on}}$, which is defined as:

(6) $$\Delta d_{i-D}^{\mathrm{off},\mathrm{on}}\equiv \frac{d_{i-D}^{\mathrm{off}}-d_{i-D}^{\mathrm{on}}}{d_{i-D}^{\mathrm{on}}}\times 100\%$$

**Figure 6:**
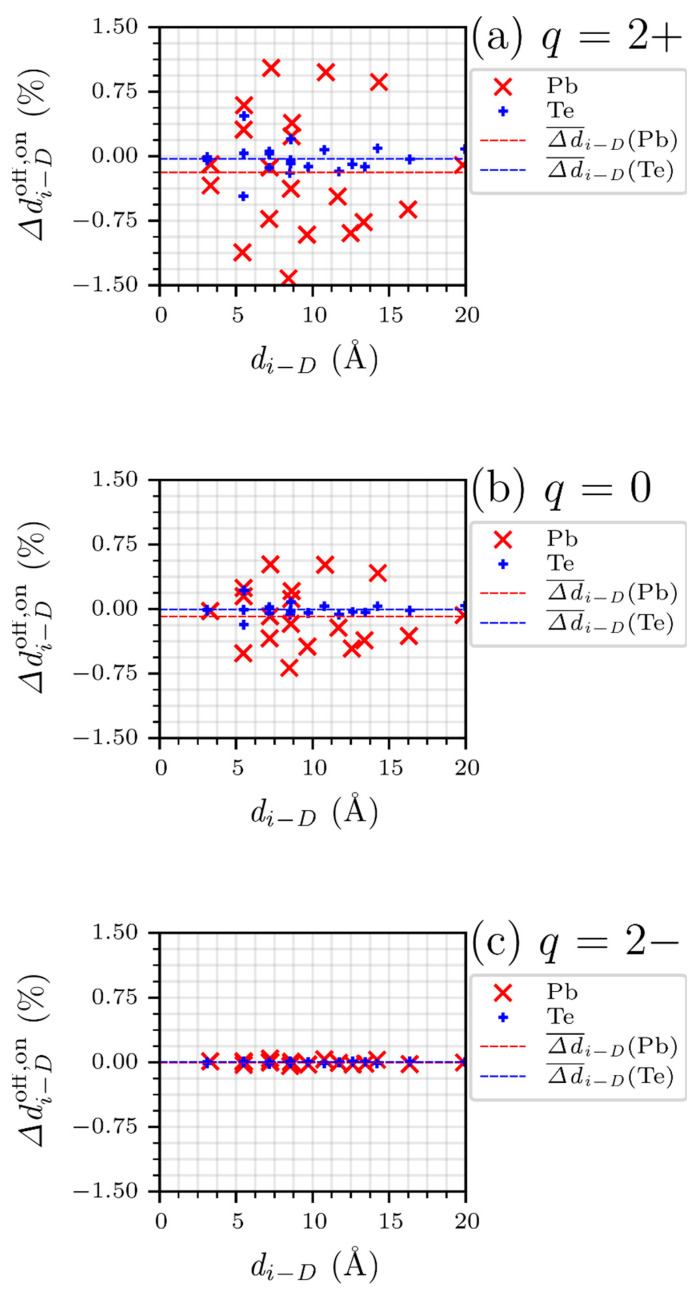
Structure-contrast parameter $\Delta d_{i-D}^{\mathrm{off},\mathrm{on}}$ of Pb (red ×) and Te (blue +) under the charge state of (**a**) 2+, (**b**) neutral, and (**c**) 2−. The red and blue dashed lines represent the average of $\Delta d_{i-D}^{\mathrm{off},\mathrm{on}}$ for Pb and Te, respectively. The x-axis represents distance parameter $d_{i-D}$.

From the data of $\Delta d_{i-D}^{\mathrm{off},\mathrm{on}}$ in Figure 6, the structural relaxation of Pb is much larger than that of Te regardless of the charge state, except for the 2− charge state. This is consistent with the results in Figure 5, where the introduction of $I_{\mathrm{Pb}}$ has a greater effect on Pb than on Te in its supercell. In addition, as the charge state changes from positive to negative, the overall $\Delta d_{i-D}^{\mathrm{off},\mathrm{on}}$ clearly decreases, indicating that the off-centered defect configuration reverts to the on-centered one. It also indicates that $I_{\mathrm{Pb}}$ has a greater effect on the atomic structure of the PbTe lattice when its charge state changes from negative to positive, which is consistent with the charged defect formation energy (i.e., the energy reduction by breaking the symmetry is significant for the 2+ charge state but vanishes for the 2− state).

Then, the effect of $I_{\mathrm{Pb}}$ on PbTe lattice distortion was investigated by computing the atomic distances between each host Pb and its SNN Te because Pb or Te inside PbTe experiences structural changes due to $I_{\mathrm{Pb}}$. Note that the SNN Te is in the [111] direction or other equivalent directions from each host Pb in PbTe. Because supercells containing each of the three types of $I_{\mathrm{Pb}}$ have different structural features, distinct strain effects from the corresponding $I_{\mathrm{Pb}}$ are expected. To verify this, the structural distortion parameter along the [111] direction was calculated, defined as:

(7) $${\bar{d}}_{\mathrm{Te}-\mathrm{Pb}}^{\mathrm{SNN}}\equiv \frac{1}{N_{\mathrm{Pb}}}\sum_{\mathrm{Pb}}\left(\frac{\sum^{\;}d_{\mathrm{Te}-\mathrm{Pb}}^{\mathrm{SNN}}}{N_{\mathrm{SNN}}^{\mathrm{Te}}}\right)$$

for all host Pb atoms in each defective supercell. $d_{\mathrm{Te}-\mathrm{Pb}}^{\mathrm{SNN}}$ is the distance from one Pb atom to its SNN Te atom in the host PbTe, $N_{\mathrm{SNN}}^{\mathrm{Te}}$ is the total number of SNN Te of all host Pb atoms in the PbTe supercell, and $N_{\mathrm{Pb}}$ is the total number of host Pb atoms. Pristine PbTe has a fixed $d_{\mathrm{Te}-\mathrm{Pb}}^{\mathrm{SNN}}$ of 5.695 Å. In Table 2, all ${\bar{d}}_{\mathrm{Te}-\mathrm{Pb}}^{\mathrm{SNN}}$ values of the supercell involving $I_{\mathrm{Pb}}$ are smaller than that of pristine PbTe. Furthermore, ${\bar{d}}_{\mathrm{Te}-\mathrm{Pb}}^{\mathrm{SNN}}$ for atoms far from the defects, that is, $d_{i-D}>$ 4.886 Å, significantly decreases in the order of $I_{\mathrm{Pb}}^{\mathrm{on},2+}$, $I_{\mathrm{Pb}}^{\dim,2+}$, and $I_{\mathrm{Pb}}^{\mathrm{off},2+}$. Note that owing to the stronger interaction between $I_{\mathrm{Pb}}$ and the host atoms with $d_{i-D}\leq$ 4.886 Å, including only FNN atoms of $I_{\mathrm{Pb}}$, results in a smaller ${\bar{d}}_{\mathrm{Te}-\mathrm{Pb}}^{\mathrm{SNN}}$ than when those are excluded. This is consistent with our finding in Table 1 that atoms in the FNN shell of $I_{\mathrm{Pb}}$ experience larger position changes than the other atoms. The distances between $I_{\mathrm{Pb}}$ and its FNN atoms are smaller than 4.886 Å for all supercells. Moreover, even when excluding the FNNs of $I_{\mathrm{Pb}}$, ${\bar{d}}_{\mathrm{Te}-\mathrm{Pb}}^{\mathrm{SNN}}$ decreases in the order of $I_{\mathrm{Pb}}^{\mathrm{on},2+}$, $I_{\mathrm{Pb}}^{\dim,2+}$, and $I_{\mathrm{Pb}}^{\mathrm{off},2+}$, which reflects the order of the average $R_{i}\left(D\right)$ of Pb and Te inside the host PbTe (see Figure 5). As a result, owing to smaller distances between Pb and SNN Te, each Pb and its SNN Te in the host PbTe can form a weak local dipole, indicating that $I_{\mathrm{Pb}}$-induced ferroelectric-like domains can occur inside the host supercell. In particular, $I_{\mathrm{Pb}}^{\mathrm{off},2+}$ may induce a stronger ferroelectric-like domain than the other interstitials.

From the above results, it is concluded that the structural change caused by interstitial defects is significant for atoms far from the defects. This structural change is prominent in the [111] direction, which is the direction of the off-center displacement of Pb interstitial defects. Finally, it was investigated whether Pb interstitial defects induce ferroelectric-like phase transformations in our supercell of Pb_65_Te_64_. For this, the degree of ferroelectric-like phase transformation in the PbTe with interstitial defects was obtained by computing the average structural change in Pb or Te atoms. Here, we do not allow the lattice dynamics of defective supercells, but only the atomic structural relaxations. Without interstitial defects, the Pb and Te atoms are at the (0 0 0) and (1/2 1/2 1/2) positions, while with interstitial defects these positions change to (δ δ δ) and (1/2 1/2 1/2), respectively, where δ is 0.018 for the supercell of Pb_65_Te_64_, which is comparable to δ = ~0.023 for ferroelectric GeTe [56,57,58] with *a* = 6.00 Å. With a larger δ for PbTe, it is expected that allowing lattice distortion will enhance the structural distortion.

The off-centered $I_{\mathrm{Pb}}$ significantly affects atomic positions in the host lattice. Because the interaction is long-range, atomic structure relaxation was observed at a distance of ~1 nm from the defect site. Moreover, most Pb and Te atoms in the host PbTe are displaced along the $\left[\bar{1}\bar{1}\bar{1}\right]$ and [111] directions to form a rhombohedral-like structure. With respect to the emphanisis of PbTe [27,28,32,59,60], the temporal or spatial average positions of host Pb atoms in pristine PbTe are at the rock-salt lattice points. However, it is expected that the existence of $I_{\mathrm{Pb}}$ permanently moves the host Pb atoms with a specific displacement along the direction of its SNN Te.

Our results also demonstrate that symmetry breaking of defects leads to defect–lattice interactions. The lattice parameter of PbTe used here is similar to the experimentally measured value at 900 K and larger than the experimental room-temperature value. Hence, our results reflect the high-temperature energetics for intrinsic defects along with a defect–lattice interaction analysis in PbTe. In contrast to the phase transition of GeTe from cubic to rhombohedral structures as the temperature changes from high to low [56,57,58], PbTe is expected to experience a local phase transition from cubic to rhombohedral structures at temperatures above 900 K by forming $I_{\mathrm{Pb}}^{\mathrm{off}}$.

Finally, our calculation clearly shows that $I_{\mathrm{Pb}}^{\mathrm{off}}$ can form in four potential directions, and the resulting defect lattice interactions can affect PbTe in a range exceeding 1 nm. If $I_{\mathrm{Pb}}^{\mathrm{off}}$ is formed in different directions inside PbTe, the material can possess ferroelectric-like rhombohedral domains or emphanitic regions due to $I_{\mathrm{Pb}}^{\mathrm{off}}$ aligned in different directions. Just as the lattice thermal conductivity of GeTe decreases due to domain boundaries formed by the differently aligned herringbone structures [61], Pb-rich PbTe can have similar domain boundaries due to $I_{\mathrm{Pb}}^{\mathrm{off}}$. Because the domain boundaries are caused by the rhombohedral domains or emphanitic regions, they may lead to a decrease in lattice thermal conductivity, which could be additionally responsible for the intrinsically low lattice thermal conductivity of binary PbTe. The likely reason is that a phonon blocking barrier is formed at the boundaries between ${\mathrm{Pb}}_{\mathrm{Int}}$-induced ferroelectric-like domains, in addition to the strongly anharmonic nature of phonon in PbTe.

## 4. Conclusions

DFT calculations were performed to locate the low-symmetry off-centered Pb interstitial defects with lower defect formation energies in PbTe. It was found that the off-centered Pb interstitials are multi-stable defects while the on-centered defects are unstable. A saddle Pb–Pb dimeric interstitial structure was also identified. Owing to the lower formation energy of the multi-stable off-centered defect, our calculated defect density is larger than those reported previously. Structural analysis revealed that structural distortion along the [111] direction is significant for the 2+ charge state and reduced in the neutral and negatively charged states. In contrast to the on-centered defects, the off-centered ones exhibit long-range structural relaxation effects, which might be responsible for the local rhombohedral phase transformation. We believe that the intrinsic off-centering of Pb interstitials is another possible mechanism for the low thermal conductivity of PbTe at high temperatures.

## Figures and Tables

**Table 1:** Distributions of $d_{i-D}^{\left(0\right)}$, $R_{i}$, $d_{i-D}$, and the number of atoms (#) for the host Pb (top part) and Te (bottom part) atoms in the defective supercell with the on-centered ($I_{\mathrm{Pb}}^{\mathrm{on},2+}$) and off-centered ($I_{\mathrm{Pb}}^{\mathrm{off},2+}$) Pb interstitial defects, where $d_{i-D}^{\left(0\right)}\leq 7.166\;Å$. FNN, SNN, and TNN mean the first-, second-, and third-nearest neighbors, respectively.

Host Pb	$d_{i-D}^{\left(0\right)}$ (Å)	$I_{Pb}^{on,2+}$			$I_{Pb}^{off,2+}$		
		$R_{i}$ (Å)	$d_{i-D}$ (Å)	#	$R_{i}$ (Å)	$d_{i-D}$ (Å)	#
FNN	2.847	0.502	3.349	4	0.431	3.337	1
					0.518	3.346	3
SNN	5.452	0.048	5.500	12	0.046	5.438	3
					0.048	5.516	6
					0.092	5.532	3
TNN	7.166	0.060	7.225	12	0.053	7.172	3
					0.081	7.215	6
					0.079	7.299	3
**Host Te**	$d_{i-D}^{\left(0\right)}$ **(Å)**	$I_{Pb}^{on,2+}$			$I_{Pb}^{off,2+}$		
		$R_{i}$. **(Å)**	$d_{i-D}$ **(Å)**	#	$R_{i}$ **(Å)**	$d_{i-D}$ **(Å)**	#
FNN	2.847	0.260	3.108	4	0.257	3.106	3
					0.319	3.108	1
SNN	5.452	0.061	5.512	12	0.055	5.486	3
					0.115	5.514	6
					0.098	5.538	3
TNN	7.166	0.016	7.181	12	0.062	7.171	3
					0.077	7.183	6
					0.077	7.185	3

**Table 2:** Values of ${\bar{d}}_{\mathrm{Te}-\mathrm{Pb}}^{\mathrm{SNN}}$ of host Pb atoms with $d_{i-D}$ ≤ 4.886 Å or > 4.886 Å in supercells with the Pb interstitial defects of $I_{\mathrm{Pb}}^{\mathrm{on},2+}$, $I_{\mathrm{Pb}}^{\mathrm{off},2+}$, and $I_{\mathrm{Pb}}^{\dim,2+}$.

**Supercell Type**	${\bar{d}}_{Te-Pb}^{SNN}$ **(Å)**	
	$d_{i-D}\leq$4.886 (Near Defect)	$d_{i-D}>$ 4.886 (Far from Defect)
$I_{\mathrm{Pb}}^{\mathrm{on},2+}$	5.519	5.670
$I_{\mathrm{Pb}}^{\mathrm{off},2+}$	5.521	5.630
$I_{\mathrm{Pb}}^{\dim,2+}$	5.551	5.660

## Data Availability

The data that support the findings of this study are available from the corresponding author upon reasonable request.
